# Intermittent Hypobaric Hypoxic Preconditioning Provides Neuroprotection by Increasing Antioxidant Activity, Erythropoietin Expression and Preventing Apoptosis and Astrogliosis in the Brain of Adult Rats Exposed to Acute Severe Hypoxia

**DOI:** 10.3390/ijms22105272

**Published:** 2021-05-17

**Authors:** Débora Coimbra-Costa, Fernando Garzón, Norma Alva, Tiago C. C. Pinto, Fernando Aguado, Joan Ramon Torrella, Teresa Carbonell, Ramón Rama

**Affiliations:** 1 Department of Cellular Biology, Physiology and Immunology, Faculty of Biology, Universitat de Barcelona, Av. Diagonal 643, 08028 Barcelona, Spain; fisiodeby@yahoo.com.br (D.C.-C.); garzongomezfernando@gmail.com (F.G.); nvalva@ub.edu (N.A.); faguado@ub.edu (F.A.); tcarbonell@ub.edu (T.C.); rrama@ub.edu (R.R.); 2 Department of Neurophychiatry and Behavioural Science, Universidade Federal de Pernambuco, Av. da Engenharia, 186-298, Cidade Universitaria, Recife 50740-600, PE, Brazil; tcoimbra.pinto@gmail.com

**Keywords:** apoptosis-inducing factor, erythropoietin, glial fibrillary acidic protein, glutathione, HIF, NF-κB, superoxide dismutase

## Abstract

Background: Exposure to intermittent hypoxia has been demonstrated to be an efficient tool for hypoxic preconditioning, preventing damage to cells and demonstrating therapeutic benefits. We aimed to evaluate the effects of respiratory intermittent hypobaric hypoxia (IHH) to avoid brain injury caused by exposure to acute severe hypoxia (ASH). Methods: biomarkers of oxidative damage, mitochondrial apoptosis, and transcriptional factors in response to hypoxia were assessed by Western blot and immunohistochemistry in brain tissue. Four groups of rats were used: (1) normoxic (NOR), (2) exposed to ASH (FiO_2_ 7% for 6 h), (3) exposed to IHH for 3 h per day over 8 days at 460 mmHg, and (4) ASH preconditioned after IHH. Results: ASH animals underwent increased oxidative-stress-related parameters, an upregulation in apoptotic proteins and had astrocytes with phenotype forms compatible with severe diffuse reactive astrogliosis. These effects were attenuated and even prevented when the animals were preconditioned with IHH. These changes paralleled the inhibition of NF-κB expression and the increase of erythropoietin (EPO) levels in the brain. Conclusions: IHH exerted neuroprotection against ASH-induced oxidative injury by preventing oxidative stress and inhibiting the apoptotic cascade, which was associated with NF-κB downregulation and EPO upregulation.

## 1. Introduction

The mammalian brain requires a continuous supply of oxygen to maintain its structure and properly develop its function. When undergoing hypoxia, brain cells may remodel, became injured or die depending on the severity and duration of the hypoxic insult. Thus, the brain cannot tolerate a significant reduction in partial pressure of oxygen (PO_2_) for long periods due to the inability of the anaerobic glycolysis metabolic pathway to provide enough energy supply [1]. Since only the oxidative metabolism can provide the high-energy demands required by brain cells, a continuous supply of oxygen and glucose is needed, making the brain a very sensitive organ PO_2_ reductions, such as in conditions of respiratory hypoxia [2,3]. However, sublethal hypoxic conditions stimulate neurogenesis and angiogenesis by the secretion of trophic factors [4], as has been described during embryonic brain development, where oxygen concentrations ranging from 1% to 5% allow the CNS cells to differentiate and proliferate, indicating that under certain circumstances, hypoxia plays an important role in neurogenesis, neuroprotection and neuronal survival [5]. In fact, it is known that brief hypoxia exposures can elicit neuroprotection by enhancing the tolerance of the exposed organism to ischemia [6] or severe hypoxia (see for review [7]). Brain preconditioning is a phenomenon by which repeated doses of a stressful sublethal stimulus protect the brain against subsequent injury. Intermittent hypoxia, i.e., repeated episodes of hypoxia interspersed with episodes of normoxia, is a form of preconditioning widely used to induce neuroprotection against severe hypoxia and ischemia [8]. Although both in hypoxia and ischemia, oxygen deficiency is a common factor, ischemia is a hypoxic episode characterized by insufficient nutrient supply due to decreased perfusion. In contrast, respiratory hypoxia involves decreased oxygen concentration in the tissue without affecting blood flow and nutrient supply [9]. The effects of preconditioning on the expression of antioxidants and apoptosis-related factors have been studied extensively in cerebral ischemia (see, for example, [10,11]), but few studies have addressed the effects of hypoxic preconditioning after respiratory hypobaric hypoxia in the brain [12,13,14,15]. Hypoxic preconditioning and its involved adaptive factors are interesting because hypoxia is at the base of the pathogenesis of many diseases, and preventing damage to cells by inducing milder processes that cause it could have several therapeutic benefits. Several uses of hypoxic preconditioning have been proposed. For example, to protect the neonatal brain, which is susceptible to hypoxic–ischemic injury due to its developmental characteristics [16]; to act against ischemic cell death that takes place in stroke [17], or to be used in preoperative interventions [9].

Hypoxic preconditioning could induce its neuroprotective action by triggering several mechanisms, such as enhancing vascular regulation, the activation of antiapoptosis and antioxidant signaling pathways, the suppression of excitotoxicity and the promotion of cell proliferation [16]. The hypoxia-inducible factor (HIF) has emerged as the master transcriptional regulator of oxygen homeostasis, controlling the expression of multiple genes, including those that encode erythropoietin (EPO) and other proteins involved in red blood cell production and, thus, increasing blood oxygen-carrying capacity that may contribute to hypoxia-induced tolerance [18]. Moreover, over the last few years, a neuroprotective role for EPO has been described. It has been shown that in neurons, the binding of EPO to its receptor leads to the activation of several signaling pathways ending in transcription factors that move to the nucleus and initiate the expression of Bcl-2 and Bcl-xL genes [19]. These proteins play a pivotal role in counteracting the activation of proapoptotic proteins by regulating the permeabilization of the mitochondrial outer membrane and consequently the release of cytochrome *c* into cytoplasm and the mitochondrial intrinsic apoptotic pathway [20]. The response to hypoxia is not only based on HIF since many other transcription factors have shown a response to hypoxia [21]. Among these is the transcription factor family Nuclear Factor-κB (NF-κB) formed by five different proteins that can diversely combine to form an active transcriptional dimer implicated in regulating cellular proliferation, inflammatory response, cell apoptosis and cell survival [22,23]. Oxidative stress that results from hypoxia exposure also plays an important role in the activation of NF-κB [21,24]. Recently, some evidence has been provided about the function of astrocytes as specialized CNS sensors for detecting physiological decreases in brain oxygenation [25]. The detection of low PO_2_ by these cells would stimulate the neurons that contribute to the respiratory response to hypoxia and increase the oxygenation of the arterial blood. Additionally, astrocytes are believed to release vasoactive substances, significantly affecting cerebral vasculature by controlling local cerebral microcirculation at low PO_2_ [26].

The present work aimed to evaluate the effects of an intermittent hypobaric hypoxia preconditioning protocol (4 h/day for 8 days at 460 mmHg) on brain injury caused by exposure to acute severe normobaric hypoxia (6 h at 7% FiO_2_), analyzing the pro-oxidant and antioxidant system, mitochondrial apoptotic markers, levels of hypoxia transcriptional factors, EPO and astrocyte’s morphology and reactivity in the brains of rats.

## 2. Results

### 2.1. Oxidative Stress

**Figure 1:**
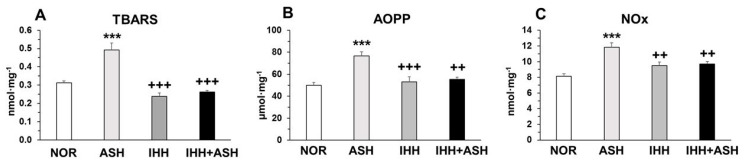
Oxidative stress markers in the brain. Units are expressed per milligram of protein. (**A**) TBARS, thiobarbituric acid-reactive substances; (**B**) AOPP, advanced oxidation protein products; and (**C**) NOx, nitric oxide species. Statistically significant differences between groups are presented with asterisks (*, vs. NOR) or crosses (+, vs. ASH). Two symbols indicate *p* < 0.01 and three symbols *p* < 0.001. F-values after running one-way ANOVAs with Bonferroni’s post hoc multiple comparison tests were: F = 10.83 (TBARS), F = 12.42 (AOPP) and F = 6.33 (NOx). Animal experimental groups are abbreviated as NOR, normoxic; ASH, acute severe hypoxia; IHH, intermittent hypobaric hypoxia; IHH+ASH, intermittent hypobaric hypoxia followed by acute severe hypoxia.

Exposure to ASH generated increased oxidative stress that led to significantly (*p* < 0.001) higher levels of lipid (thiobarbituric acid reactive substances, TBARS) and protein oxidation (advanced oxidation protein products, AOPP) and nitric oxide species (NOx) (Figure 1). This increase was prevented in IHH and IHH+ASH, showing a statistically significant reduction compared to ASH. Antioxidant enzymes, such as superoxide dismutase (SOD) and reduced glutathione (GSH), decreased significantly in ASH and recovered in both groups exposed to intermittent hypoxia (Table 1). Reduced glutathione (GSH) decreased significantly in all hypoxia-exposed groups, and oxidized glutathione (GSSG) showed no statistical differences between the groups. However, the greater significant decrease in GSH combined with the non-significant increase in GSSG resulted in a statistically significant decrease (*p* < 0.001) in GSH/GSSG ratio in ASH. This ratio did not show significant differences either in IHH or in IHH+ASH when compared to NOR. Glutathione peroxidase (GPx) was reduced in all the hypoxic groups, especially in those exposed to intermittent hypoxia, which presented statistically significant differences not only to NOR but also to ASH. Glutathione reductase (GR) decreased significantly due to the ASH insult, which was prevented both in IHH and IHH+ASH groups.

### 2.2. Mitochondrial Apoptosis

**Figure 2:**
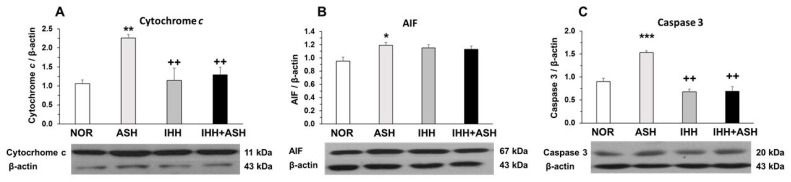
Mitochondrial apoptosis-related markers. Examples of Western blots bands normalized to β-actin (with each corresponding well) are shown. Units are expressed relative to the β-actin content. (**A**) cytochrome *c*; (**B**) AIF, apoptosis-inducing factor; (**C**) caspase-3. Statistically significant differences between groups are presented with asterisks (*, vs. NOR) or crosses (+, vs. ASH). One symbol indicates *p* < 0.05, two symbols *p* < 0.01 and three symbols *p* < 0.001. F-values after running one-way ANOVAs with Bonferroni’s post hoc multiple comparison tests were: F = 4.29 (cytochrome *c*), F = 2.93 (AIF) and F = 1.61 (caspase 3). Animal experimental groups are abbreviated as NOR, normoxic; ASH, acute severe hypoxia; IHH, intermittent hypobaric hypoxia; IHH+ASH, intermittent hypobaric hypoxia followed by acute severe hypoxia.

The measured mitochondrial apoptosis indicators, i.e., cytochrome *c*, apoptosis-inducing factor (AIF) and active caspase-3, showed significant increases in ASH, as indicated by Western blot semi-quantification (Figure 2). However, cytochrome *c* (Figure 2A) and active caspase-3 (Figure 2C) expression were similar to NOR levels in both IHH and IHH+ASH groups, having statistically significant lower levels when compared to ASH. The slight decrease in AIF was not statistically significant (Figure 2B).

### 2.3. Transcriptional Factors in Response to Hypoxia

**Figure 3:**
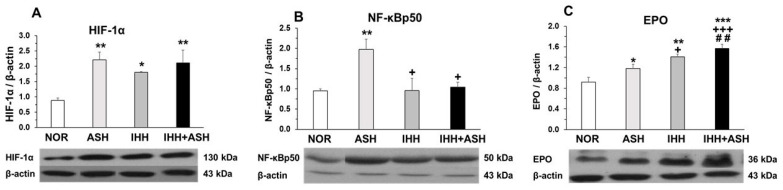
Transcriptional factors in response to hypoxia. Examples of Western blots bands normalized to β-actin (with each corresponding well) are shown. Units are expressed relative to the β-actin content. (**A**) HIF-1α, hypoxia-inducible factor 1-alpha; (**B**) NF-κBp50, nuclear factor kappa-light-chain-enhancer of activated B cells; (**C**) EPO, erythropoietin. Statistically significant differences between groups are presented with asterisks (*, vs. NOR), crosses (+, vs. ASH) or hashtags (#, vs. IHH). One symbol indicates *p* < 0.05, two symbols *p* < 0.01 and three symbols *p* < 0.001. F-values after running one-way ANOVAs with Bonferroni’s post hoc multiple comparison tests were: F = 3.96 (HIF-1α), F = 1.01 (NF-κBp50) and F = 11.98 (EPO). Animal experimental groups are abbreviated as NOR, normoxic; ASH, acute severe hypoxia; IHH, intermittent hypobaric hypoxia; IHH+ASH, intermittent hypobaric hypoxia followed by acute severe hypoxia.

Similarly, the expression of HIF-1α significantly increased in the brain of all the groups exposed to hypoxia compared to the normoxic group (Figure 3A). The expression of the phosphorylated p50 subunit of NF-κB also increased significantly in ASH (Figure 3B), but, in contrast to what was found in HIF-1α, the expression of NF-κBp50 in intermittent hypoxia-exposed groups did not differ from NOR, both groups presenting significant differences when compared to ASH.

### 2.4. Erythropoietin (EPO) Expression in the Brain

Figure 3C shows the EPO levels in the brains of the four groups of rats. It can be seen that all hypoxia-exposed animals had higher statistically significant concentrations of EPO. The greatest EPO response to hypoxia was observed in IHH+ASH, which showed an additive effect to IHH and ASH exposure. Statistically significant differences were observed between all groups showing an accumulative effect from ASH, IHH to IHH+ASH.

### 2.5. Astrocytes

**Figure 4:**
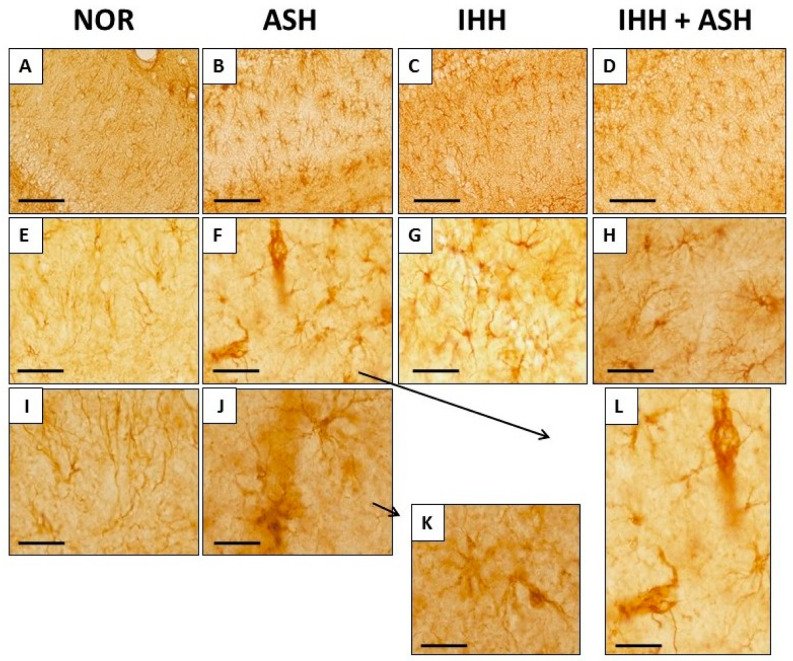
Glial fibrillary acidic protein (GFAP) immunohistochemistry in the *Cornu ammonis* (CA1) hippocampal zone at different magnifications. The increase in GFAP expression and changes in astrocyte morphology is more pronounced in ASH (**B**,**F**,**J**,**K**,**L**). Animals exposed to IHH had greater GFAP expression but did not show astrogliosis (**C**,**D**,**G**,**H**). Bars represent the following distances: ((**A**)–(**D**)), 100 µm; ((**E**)–(**H**)), 40 µm; (**I**,**J**), 20 µm; (**K**,**L**), 15 µm. Animal experimental groups are abbreviated as NOR, normoxic; ASH, acute severe hypoxia; IHH, intermittent hypobaric hypoxia; IHH+ASH, intermittent hypobaric hypoxia followed by acute severe hypoxia.

**Figure 5:**
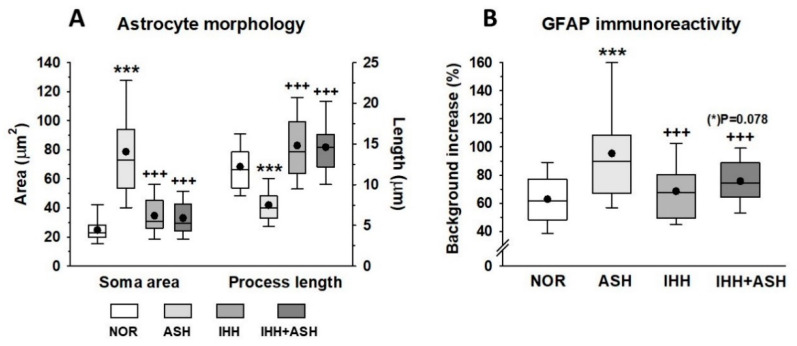
(**A**) Astrocyte morphometrical parameters (soma area and process length morphology). (**B**) expression of astrocyte glial fibrillary acidic protein (GFAP) immunoreactivity. All data were obtained in the *Cornu ammonis* (CA1) hippocampal zone. The box represents the interquartile range in the box-and-whisker plots and shows the first and third quartiles separated by the median. The black dot represents the mean, and whisker endpoints represent the minimum and maximum values. Statistically significant differences between groups are presented with asterisks (*, vs. NOR) or crosses (+, vs. ASH). The three symbols indicate *p* < 0.001. F-values after running one-way ANOVAs with Bonferroni’s post hoc multiple comparison tests were: F = 85.94 (soma area), F = 119.93 (process length) and F = 20.77 (GFAP immunoreactivity). Animal experimental groups are abbreviated as NOR, normoxic; ASH, acute severe hypoxia; IHH, intermittent hypobaric hypoxia; IHH+ASH, intermittent hypobaric hypoxia followed by acute severe hypoxia.

Figure 4 shows representative immunohistochemical slides from the *Cornus ammonis* CA1 hippocampus region stained for glial fibrillary acidic protein (GFAP). The quantitative analysis of the astrocyte morphology (soma areas and processes length) and the intensity of GFAP immunoreactivity are summarized in Figure 5. Exposure to acute severe respiratory hypoxia caused a severe diffuse reactive astrogliosis in ASH. Astrocytes from this group evidenced significant changes in their morphology with hypertrophy of the cellular bodies (larger soma areas) and retraction of the cellular extensions (shorter cell processes length) when compared to NOR astrocytes (Figure 4F,J–L and Figure 5A). Moreover, ASH astrocytes had a significant increase in GFAP protein immunoreactivity (Figure 5B). These morphological changes and immunoreactivity patterns did not appear in animals exposed to intermittent hypobaric hypoxia. In these animals, the soma areas (Figure 5A) and the expression of astrocyte GFAP immunoreactivity were significantly decreased (Figure 5B) together with a proliferation of the cellular processes compared with NOR (Figure 4 and Figure 5A). Thus, preconditioning by intermittent hypoxia (IHH+ASH) reduced the astrogliosis intensity caused by severe respiratory hypoxia compared to (ASH).

## 3. Discussion

We investigated the effects on the brain of a preconditioning protocol of exposure to intermittent hypobaric hypoxia before an acute severe normobaric hypoxia insult. In summary, the results show that: (1) exposure to ASH caused oxidative stress in the brain, as demonstrated by significant increases in lipid and protein oxidation and the NOx levels; a drop in the antioxidant capacity, as indicated the significant decrease in SOD activity, GSH levels and GSH/GSSG ratio; and activated the mitochondrial apoptotic pathway, as shown by the significant boost of cytochrome *c*, AIF and caspase-3 expression; (2) a protocol of IHH before exposure to ASH prevented these deleterious effects avoiding oxidative stress and maintaining the antioxidant capacity and the apoptotic markers at baseline (NOR) conditions; (3) the expression of HIF-1α and EPO in the brain of all groups exposed to hypoxia increased significantly when compared to NOR, both acute-exposed and preconditioned animals presenting similar values for HIF-1α, but the preconditioned group (IHH+ASH) having significantly higher EPO expression than the ASH- or IHH-exposed groups; (4) in contrast to this behavior, the significant increase found in NF-κB levels after ASH exposure was prevented by the IHH protocol; (5) ASH induced in hippocampus astrocytes’ morphology changes compatible with severe diffuse reactive astrogliosis, while IHH+ASH reduced the astrogliosis intensity to mild to moderate and increased GFAP expression compared to NOR.

### 3.1. Oxidative Stress

Brains from rats exposed to severe hypoxia (ASH) increased their oxidant activity leading to the oxidation of lipids (TBARS), proteins (AOPP) and nitric oxide species (NOx) (Figure 1). This was concomitant with decreased antioxidant activity as the decrease of SOD and GR activities and the ratio GSH/GSSG indicated (Table 1). All these results conform to a situation of oxidative distress [27] and are coincident with previous results from our group [28] and with findings reported by others [29,30,31].

Under normoxic conditions, the combined action of antioxidants and oxidants maintains the homeostasis of free radicals. Since cell survival depends on strict regulation of the free radical content, an excess of free radicals leads to a situation of oxidative distress, while a deficit of free radicals (specifically hydrogen peroxide) leads to oxidative eustress, which may affect important cellular signaling pathways [32]. Neurons obtain ATP primarily through oxidative phosphorylation via the mitochondrial electron transport chain, a process where it is a continuous production of the radical superoxide (O_2_^−^). This radical is maintained within homeostatic levels by antioxidant systems, such as SOD [33,34]. Severe hypoxia affects the process of mitochondrial oxidative phosphorylation leading to increased production of O_2_^−^ [35] and decreased antioxidant capacity elicited by SOD and GR [36]. SOD functions keeping the O_2_^−^ homeostatic levels generating H_2_O_2,_ which is converted to H_2_O by oxidation of GSH to GSSG through a concerted cascade of mechanisms involving GPx and GR [37]. Our results show that exposure to severe hypoxia (ASH) caused decreased SOD and GSH and increased GSSG, resulting in a decreased GSH/GSSG ratio (Table 1). Under normoxic conditions, this ratio is maintained by the action of GR and is decreased in hypoxia [29]. Thus, the increased production of reactive oxygen species (ROS) coupled with the decrease in SOD observed in our study in ASH implies that a fraction of the O_2_^−^ is not reduced to H_2_O_2_. In the same way, the decreased levels of GSH may be insufficient to reduce the H_2_O_2_ to H_2_O, leading to a buildup of H_2_O_2_. These conditions derive from an accumulation of O_2_^−^ and H_2_O_2,_ which can, respectively, react either with NO^−^, forming peroxynitrite (ONOO^−^) or with ferrous ion (Fe^2+^) rendering ^−^OH and Fe^3+^ (Fenton reaction) [38]. Both ONOO^−^ and ^−^OH are highly reactive radicals, which explain the presence of oxidized forms of lipids and proteins that we have found in the brains of the ASH rats (Figure 1).

Our results show that the above-mentioned oxidative stress caused by the acute exposure to severe hypoxia was inhibited when the animals were previously exposed to a protocol of intermittent hypobaric hypoxia. IHH+ASH animals had a level of oxidation of lipids and proteins and the presence of NOx similar to the NOR group, with significantly lower values than ASH (Figure 1). Moreover, the antioxidant activity of SOD, GR, and the ratio GSH/GSSG did not differ from those observed in the brains of NOR rats (Table 1), indicating that the preconditioning protocol of intermittent hypoxia had a neuroprotective effect by preventing oxidative distress. Similar results were obtained by Stroev et al. [15] using a shorter hypoxic preconditioning protocol and exposing the animals to a more severe acute hypoxia. However, the present study additionally shows that preconditioning elevated SOD levels without a period of reoxygenation after IHH+ASH. As a future perspective to confirm these findings, it would be interesting to analyze protein or mRNA abundance of catalase and peroxidase since ROS damage caused by H_2_O_2_ can be mitigated by these enzymes. Additionally, to analyze some electron transport chain complex subunits (e.g., complex I or complex III) would also be particularly interesting due to the high mitochondrial sensibility to hypoxia.

There is concluding evidence that oxidative stress is linked to several neuropathological processes involving specific mitochondrial targets [39], suggesting that the mitochondria could be the main cell sites where brain damage produced by severe acute hypoxia exposure would act. In fact, under hypoxic conditions, mitochondria are the major source of ROS [40,41], and it is well-known that hypoxia alters mitochondrial function by affecting the oxidative phosphorylation due to decreased electron transport rate, a reduction of the mitochondrial membrane potential (∆ψm), and an enhanced nitric oxide synthase [42,43,44]. Furthermore, the mitochondria can be targeted by their own radicals [45], and in turn, ROS and reactive nitrogen species can interact to form peroxynitrite, affecting the function of key enzymes, such as mitochondrial complex I and resulting in less ATP production [39]. This complex interplay between oxidative stress and mitochondrial dysfunction may lead to a mechanism that activates the proapoptotic cascade. Thus, we aimed to determine if preconditioning, demonstrating prevention of oxidative stress, would also prevent mitochondrial apoptosis.

### 3.2. Mitochondrial Apoptosis

Levels of mitochondrial apoptosis markers, such as cytochrome *c*, AIF and active caspase-3, showed a significant increase in the brains of rats exposed to ASH (Figure 2). In contrast, when ASH was preceded by IHH exposure (IHH+ASH), the levels of cytochrome *c* and active caspase-3 did not differ from those found in normoxic rats. This antiapoptotic behavior induced by IHH preconditioning matches the prevention of oxidative stress markers, suggesting that prevention of oxidative stress in IHH and IHH+ASH groups also avoided mitochondrial dysfunction and apoptosis. Some explanation to the neuronal protective mechanisms involved after hypoxic preconditioning could be derived from a work that analyzed immunohistochemically the patterns of expression of antiapoptotic proteins Bcl-2 and Bcl-xL in rat hippocampus and neocortex after three sessions of IHH [13], where it was concluded that the protective action was in part mediated by the shift of neuronal Bax/Bcl-2-Bcl–xL ratio.

Contrasting with the results obtained for cytochrome *c* and caspase-3, we did not find significant differences in AIF levels between IHH and IHH+ASH groups and ASH (Figure 2B), suggesting that AIF was not involved in the mechanisms of neuroprotection induced by IHH. Since AIF has been described as a neuroprotective factor in the processes of excitotoxicity [46], our results suggest that the harmful effect caused by ASH in the brain is not associated with excitotoxicity, as is in the case of ischemic hypoxia. This conclusion agrees with other studies [47,48], reinforcing the idea that, although hypoxia is the determining factor in both severe respiratory hypoxia and ischemic hypoxia, respiratory hypoxia induces different responses in the brain from those induced by ischemic hypoxia.

### 3.3. Transcriptional Factors in Response to Hypoxia

The expression of HIF-1α increased in the brain of all the groups exposed to hypoxia compared to the normoxic group (Figure 3A), with no significant differences between animals exposed to ASH and those exposed to IHH+ASH. Since ASH caused significant oxidative stress, while preconditioning with IHH did not, the expression of HIF-1α seems independent of the presence of a state of oxidative stress in the brain, although it has been described that free radicals induced by hypoxia stabilize HIF-1α [49,50]. In any case, our results indicate that HIF-1α does not play an important role in preconditioning processes induced by IHH.

NF-κB had a similar type of behavior to HIF-1α in ASH rats, presenting a significant increase in the expression of its phosphorylated p50 subunit (Figure 3B). This increased expression of NF-κBp50 in response to exposure to severe hypoxia was already observed in previous work from our laboratory [28] and reported by others [22,51]. However, contrasting with HIF-1α, in the brains of IHH-exposed rats, the expression of NF-κB did not differ from the levels found in the brains of normoxic rats. Thus, there is a different type of behavior in response to hypoxia between HIF-1α and NF-κB since IHH interferes with the activation mechanisms of the NF-κBp50 subunit, but not with those of HIF-1α expression. Overall, these results indicate that the decrease in NF-κB in the brain of IHH and IHH+ASH rats was associated with the neuroprotective effect derived from reducing oxidative stress and the apoptotic markers and was independent of the expression levels of HIF-1α. Although the mechanisms of activation of NF-κB in response to hypoxia are very complex and not well-known [22,24], our results indicate that, while the expression of NF-κB depend on the occurrence of oxidative stress [52], the expression of HIF-1α depends on the hypoxic stimulus, regardless of whether it generates oxidative stress or not. Our results contrasted with those found by others [14,53], who reported an upregulation of NF-κB in the neocortex and hippocampus after quantitative immunohistochemistry. Two reasons could afford these contrasting results. On one hand, the differences in the IHH preconditioning protocols (days of exposure and barometric pressure), the ASH “dose” received by the exposed animals (6 h and 7% FiO_2_ vs. 3 h at 5% FiO_2_) and the absence or presence of a reoxygenation period. On the other hand, the well-known dual effects as survival or a pro-death factor in the nervous system described for NF-κB (see for review [54]) could also play a role in the reported opposing findings.

### 3.4. Erythropoietin (EPO) Expression in the Brain

The EPO gene was identified as one of the genes activated by HIF-1α in response to hypoxia [55]. Initially, it was believed that the only function of EPO was to regulate the number of erythrocytes in the blood, allowing maturation of erythrocyte precursors by inhibiting apoptosis [56]. Over the past decade, it has been shown that EPO is also induced by hypoxia in the central nervous system, acting in the brain as an antiapoptotic, antioxidant, anti-inflammatory, neuroprotective and neurotrophic factor [57,58]. In this work, we have found that EPO levels were significantly increased in all groups exposed to hypoxia, matching the increases in HIF-1α (Figure 3). It is noteworthy that EPO levels were higher in IHH-exposed animals’ brains, with the highest significant differences compared to NOR in the IHH+ASH group, indicating that IHH preconditioning favored the synthesis of EPO [59]. Moreover, the IHH+ASH group showed significantly higher EPO expression than ASH and IHH. As we have suggested recently, the neuroprotective effect of EPO is associated with increases in antioxidant activity (Figure 1, Table 1) [60] and attenuates apoptosis [61] (Figure 2 and Figure 3). This demonstrates that EPO would act as a powerful antioxidant allowing the neurons to regulate their intracellular redox condition [62] and suggesting that increased EPO levels in the brain after hypoxia exposure is a potential approach for promoting neuronal survival in vivo.

EPO can accomplish its protective role by targeting components common to many different neurodegenerative pathways, which results in a plethora of different actions [63]. Mechanistically, it has been proposed that EPO action takes place through multiple signaling pathways that can lead to tissue repair and cell protection [64]. These signaling pathways increase the expression of antiapoptotic proteins of the Bcl-2 family, reducing the release and expression of the main markers of the intrinsic mitochondrial apoptotic pathway, such as cytochrome *c* and caspase-3, the main executioner of the mitochondrial apoptotic pathway [19,63]. The integrity of mitochondria allows maintaining its redox state, and, in consequence, the mitochondria ceases to be the main source of ROS, avoiding the appearance of oxidative stress. Thus, the high levels of EPO found in our study in the IHH+ASH group would justify the neuroprotective effect against oxidative stress and apoptosis that are also deduced from our results.

### 3.5. Astrocytes

Since astrocytes have been considered specialized cells in detecting the decrease in oxygen availability in the brain [25], we considered it interesting to investigate how these cells may respond to ASH and the effects that preconditioning could elicit on them. Astrocytes are the most abundant glial cells involved in virtually every function of the central nervous system, and the reciprocal interactions between glia and neurons are essential for many critical functions in health and disease in the brain [65,66,67]. Astrocytes respond to all forms of brain insults by a process commonly referred to as reactive astrogliosis, characterized by the hypertrophy of their cell bodies and cell extensions and the upregulation of intermediate filament proteins, such as glial fibrillary acidic protein (GFAP). This is a process normally associated with beneficial consequences. However, it can lead to harmful effects under specific conditions, changing their phenotype from a resting to a reactive form after a finely graded continuum of changes that range from reversible alterations in gene expression and cell hypertrophy to scar formation [67,68]. Our results show important changes in the phenotype of astrocytes characterized by an increased expression of GFAP protein in the hippocampal CA1 area in response to hypoxia (Figure 4 and Figure 5B). Moreover, hypertrophy in cell bodies and cell process length reduction was observed in ASH. In this group, astrocyte morphology is compatible with what is defined as severe diffuse reactive astrogliosis, while in IHH+ASH, astrocytes’ condition with abundant extensions could be classified as mild to moderate astrogliosis. In the hippocampus, compared to NOR animals, hypoxic preconditioning caused a phenotype of reactive forms that expressed GFAP. Since the existence of astrocytes that do not express detectable levels of GFAP has been described, our results based on the detection of this protein do not allow us to distinguish whether the IHH induced astrocyte proliferation or if it simply underwent increased GFAP expression that was not detected in NOR. However, support for astrocyte proliferation can be derived from previous work in the hippocampus dentate gyrus [69]. These authors suggested that the neuroprotective effect observed in preconditioned rats, exposed to three sessions of intermittent hypoxia and a subsequent ASH session, was stimulated by the neurogenesis observed after immunohistochemistry for NeuroD2 transcription factor.

In conclusion, hypoxic preconditioning with a protocol of intermittent hypobaric hypoxia had neuroprotective effects on acute severe hypoxia-induced oxidative injury by reducing oxidative stress and inhibiting the apoptotic cascade, which was mediated by NF-κB downregulation and EPO upregulation. Moreover, important changes were found in the phenotype of astrocytes in the hippocampal CA1 area in response to hypoxia. In animals exposed to acute severe hypoxia, these cells showed soma hypertrophy and process length reduction compatible with severe diffuse reactive astrogliosis. The protocol of hypoxic preconditioning also ameliorated this pathological astrocyte phenotype by reducing the astrogliosis to mild or moderate. 

## 4. Materials and Methods

### 4.1. Animals and Experimental Groups

Forty-four adult male Sprague-Dawley rats (Harlan Ibérica, Barcelona, Spain) weighing between 230 and 250 g were used for this study. The animals were maintained at 22 ± 1 °C with light/dark cycles of 12 h and had free access to food and water. Experimental procedures were conducted following the European Community Council Directive no. 86/609/EEC with the approval of the Ethical Institutional Committee on Animal Care and Research of the University of Barcelona. Every effort was made to reduce the number of animals and minimize animal suffering during the experiment.

**Figure 6:**
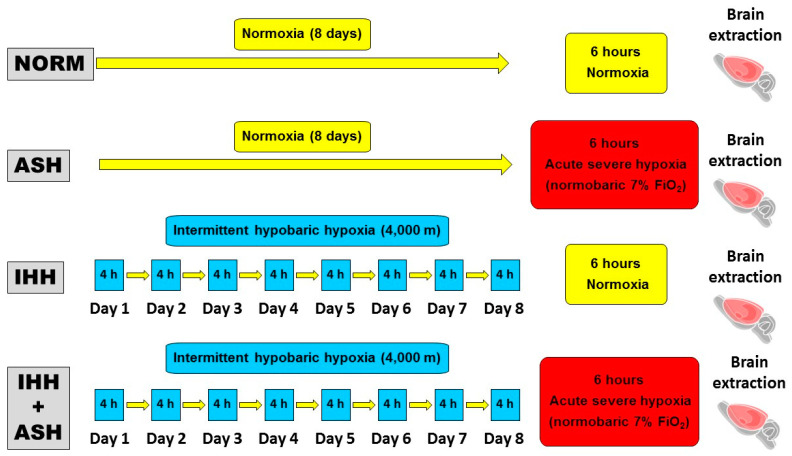
Experimental design and hypoxia exposure protocols. Animal experimental groups are abbreviated as NOR (normoxic), maintained at sea level conditions for the whole experiment; ASH (acute severe hypoxia), exposed to normobaric hypoxia at 7% O_2_ for a single 6 h continuous session; IHH (intermittent hypobaric hypoxia) exposed to hypobaric hypoxia (4000 m) for 8 consecutive days for 4 h sessions per day; and IHH+ASH, exposed to the intermittent hypobaric hypoxia protocol followed by a single session of acute normobaric hypoxia. Brain extraction was carried out after 6 h of normoxia in NOR and IHH or immediately after 6 h of acute hypoxia in ASH and HII+ASH. Yellow boxes and arrows indicate time spent in normoxia; red boxes are acute normobaric hypoxia sessions, and blue boxes time spent in hypobaric hypoxia.

Animals were randomly divided into four groups (Figure 6). (1) Group NOR (normoxic), which could be considered the control group, consisted of animals maintained in normoxic conditions (sea level) throughout the same time as the intervened animals. (2) Group ASH (acute severe hypoxia group) was comprised of animals exposed to normobaric hypoxia in a chamber with a continuous flow of a hypoxic gas mixture of 93% N_2_ and 7% O_2_ for a unique and continuous session that lasted for 6 h. ASH animals were killed by decapitation immediately after the exposure to hypoxia without giving time for reoxygenation. (3) Group IHH (intermittent hypobaric hypoxia) included animals exposed to hypobaric hypoxia for 8 consecutive days for 4 h sessions per day. A hypobaric chamber with a capacity of 136 L made of polymethyl methacrylate plastic was used to simulate an altitude of 4000 m. A relative vacuum (low-pressure) into the chamber was produced by a rotational vacuum pump (TRIVAC D5E; Leybold, Köln, Germany) by regulating a constant airflow rate of 2 L/h. Inner pressure was controlled by two differential pressure sensors (ID 2000; Leybold) connected to a vacuum controller (Combivac IT23, Leybold, Köln, Germany) driving a diaphragm pressure regulator (MR16, Leybold, Köln, Germany). (4) Group IHH+ASH consisted of rats exposed to the intermittent hypobaric hypoxia protocol followed by the exposure to the normobaric acute severe hypoxic session. After the hypoxic insult, animals were immediately killed by decapitation without giving time for reoxygenation.

Brain extraction protocol using an ice-cold isotonic saline solution (0.154 M KCl), sample storage at −80 °C and hemisphere processing for oxidative stress and Western blot analyses were completed as previously described [28].

### 4.2. Oxidative Stress Assays

#### 4.2.1. Measurement of Oxidant Activity in the Brain

Lipid peroxidation was assessed using thiobarbituric acid-reactive substances (TBARS) as indicators, following Mihara and Uchiyama [70]. Advanced oxidation protein products (AOPP) were determined spectrophotometrically according to Barsotti et al. [71], and nitric oxide species (NOx) were monitored by a colorimetric assay (Cayman Chemical, Ann Arbor, MI, USA) as described by Castillo et al. [72]. A detailed description of all these methods for measuring oxidant activity in the brain can be found in Coimbra-Costa et al. [28].

#### 4.2.2. Measurement of Antioxidant Activity in the Brain

Glutathione system. Reduced (GSH) and oxidized (GSSG) glutathione concentrations were measured in brain extracts obtained using the procedure described by [73]. Samples were homogenized in a cold 1:1 mixture (pH 6.8) of 0.1 M phosphate, 5 mM EDTA and 10% metaphosphoric acid. Later on, the homogenates were incubated in ice for 30 min, centrifuged at 100,000× *g* for 30 min, and the resulting supernatant was used to determine GSH and GSSG with the fluorescent probe ophthalaldehyde (OPA). To avoid interference, the aliquots for GSSG determination were previously incubated with N-ethylmaleimide, which reacts with GSH to produce a form that lacks luminescence. After 15 min of incubation with OPA, fluorescence was determined at 420 nm (excitation 350 nm). For determining glutathione reductase (GR) and glutathione peroxidase (GPx) activities, the tissue was homogenized by sonication in a cold buffer (pH 7.5) containing 50 mM NaH_2_PO_4_, 1 mM EDTA. Subsequently, homogenates were centrifuged at 10,000× *g* for 10 min at 4 °C. The resulting supernatant was used to measure GR and GPx activities using a Cayman kit at 340 nm (no. 703202 and no. 703102, Cayman Chemical, Ann Arbor, MI, USA).

Superoxide dismutase (SOD) activity. The brain tissue was homogenized by sonication in a cold buffer (pH 7.5) containing 50 mM NaH_2_PO_4_, 1 mM EDTA and the homogenates were centrifuged at 600× *g* for 10 min at 4 °C. SOD activity was measured from the supernatant at 450 nm using an Arbor kit (no. K028-H1, Arbor assays, Ann Arbor, MI, USA).

### 4.3. Western Blotting

The brain hemispheres were homogenized in an ice-cold lysis buffer (pH 7.4) containing 50 mM Tris-HCl, 150 mM NaCl, 1% DOC, 0.1% SDS, 1% Triton X 100, and a protease inhibitor cocktail (1:20) (sc-29130, Santa Cruz Biotechnology, Santa Cruz, CA, USA). Subsequently, the homogenates were centrifuged for 10 min at 12,000× *g,* and the proteins (50 μg) were electrophoresed and separated on 8%, 10% and 15% SDS–PAGE (Bio-Rad, Hercules, CA, USA) and transferred to nitrocellulose membranes (Whatman^®^ Schleicher & Schuell, Keene, NH, USA). Urea-SDS gels were used to improve the resolution of the proteins of low molecular weight [74]. The membranes were blocked in a solution containing 5% non-fat milk powder in a Tris-buffered saline (pH 7.6) containing 150 nM NaCl, 20 nM Tris-HCl and 0.05% Tween-20 for 1.5 h at room temperature and then thoroughly incubated with the following primary antibodies (sc: Santa Cruz Biotechnology, Santa Cruz, CA, USA): β-actin (sc-47778), cytochrome *c* (sc-13561), apoptosis-inducing factor (AIF, sc-9416), hypoxia-inducible factor 1-alpha (HIF-1α, sc-10790), nuclear factor kappa-light-chain-enhancer of activated B cells (NF-κBp50, sc-33022), and erythropoietin (EPO, sc-795216). Anti-cleaved caspase-3 antibody #9661 from Cell Signaling Technology (Danvers, MA, USA) was used to assess the expression levels of cleaved caspase-3. β-actin was used as a load control of the amount of sample protein. After several washes with TBST, the membranes were incubated for 1.5 h at room temperature, with gentle stirring, with the following horseradish peroxidase-conjugated secondary antibodies: goat anti-rabbit IgG-HRP (111-035-003 Jackson Immuno Research, West Grove, PA, USA), goat anti-mouse IgG-h+I HRP (A090-116P, Bethyl Laboratories, Montgomery, TX, USA), and rabbit anti-Goat IgG-HR (sc-2768, Santa Cruz Biotechnology, Santa Cruz, CA, USA). Finally, membranes were developed using a Thermo Scientific kit (Fisher Scientific, Waltham, MA, USA), and bands were visualized on X-ray film (Kodak, Rochester, NY, USA). Densitometric analysis was carried out using ImageJ software (NIH, Bethesda, MD, USA).

### 4.4. Immunohistochemistry

Five rats per group were anesthetized with ketamine/xylazine and fixed by intracardiac perfusion with a 0.1 M phosphate buffer (pH 7.4). The brains were obtained and frozen after cryoprotecting them in a 30% sucrose solution. They were frozen and stored until sectioned with a cryostat (Leica Microsystems, Wetzlar, Germany). Immunohistochemistry was performed as described previously [75] in coronal sections of the *Cornu ammonis* (CA1) hippocampal zone, and images were obtained with a microscope (Olympus BX-61, Tokyo, Japan). Images of slides processed for GFAP immunohistochemistry were analyzed with ImageJ (Rasband) software at 500 × magnification. The morphology of GFAP-immunoreactive astrocytes was assessed quantitatively, measuring soma areas and process lengths. The intensity of the astrocyte GFAP immune staining was expressed as a percentage increase of the background stain of each slide.

### 4.5. Statistical Analysis

Statistical Package for Social Sciences (SPSS) software (v. 13.0, IBM, Armonk, NY, USA) was used for statistical analyses. After testing normality and homoscedasticity, one-way ANOVAs, with post hoc Bonferroni’s multiple comparison tests, were run for all-group comparisons. *p*-values are given in Figures and Table as indicated in their legends.

## 5. Conclusions

This research demonstrates that intermittent respiratory hypoxia induces neuroprotective responses that prevent damage to brain cells, opening the possibility to apply hypoxic preconditioning protocols to the whole organism for therapeutic purposes.

## Figures and Tables

**Table 1:** Antioxidant activity and gluthatione system in the brain.

	NOR	ASH	IHH	IHH+ASH
**Glutathione**				
GSH (nmol·mg^−1^)	1.60 ± 0.11	1.08 ± 0.04 **	1.26 ± 0.07 *	1.19 ± 0.11 *
GSSG (nmol·mg^−1^)	0.90 ± 0.10	1.11 ± 0.06	0.88 ± 0.02	1.04 ± 0.12
GSH/GSSG	1.63 ± 0.05	0.94 ± 0.10 ***	1.43 ± 0.04	1.35 ± 0.13
**Enzymes**				
GPx (µU·mg^−1^)	15.4 ± 0.93	13.0 ± 0.49 *	8.3 ± 0.93 *** ^+++^	10.4 ± 0.62 *** ^++^
GR (µU·mg^−1^)	6.47 ± 0.50	4.28 ± 0.45 **	5.51 ± 0.28 ^+^	6.02 ± 0.53 ^++^
SOD (U·mg^−1^)	1.52 ± 0.12	1.10 ± 0.05 *	1.30 ± 0.10	1.45 ± 0.12

GPx, glutathione peroxidase; GSH, reduced glutathione; GSSG, oxidized glutathione; GR, glutathione reductase; SOD, superoxide dismutase. Values are means ± SEM. Statistically significant differences between groups are presented with asterisks (*, vs. NOR) or crosses (+, vs. ASH). One symbol indicates *p* < 0.05, two symbols *p* < 0.01 and three symbols *p* < 0.001. F-values after running one-way ANOVAs with Bonferroni’s post hoc multiple comparison tests were: F = 8.02 (GSH), F = 1.39 (GSSG), F = 11.80 (GSH/GSSG), F = 15.06 (GPx), F = 4.33 (GR) and F = 3.31 (SOD). Animal experimental groups are abbreviated as: NOR, normoxic; ASH, acute severe hypoxia; IHH, intermittent hypobaric hypoxia; IHH+ASH, intermittent hypobaric hypoxia followed by acute severe hypoxia.

## Data Availability

Not applicable.

## Abbreviations

AIF	Apoptosis-inducing factor
AOPP	Advanced oxidation protein products
ASH	Acute severe hypoxia
CA1	Hippocampus *Cornu ammonis* area 1
CNS	Central nervous system
EPO	Erythropoietin
GFAP	Glial fibrillary acidic protein
GPx	Glutathione peroxidase
GR	Glutathione reductase
GSH	Reduced glutathione
GSSG	Oxidized glutathione
HIF	Hypoxia-inducible factor
IHH	Intermittent hypobaric hypoxia
NF-κB	Nuclear factor kappa-light-chain-enhancer of activated B cells
NOR	Normoxic
NOx	Nitric oxide species
ROS	Reactive oxygen species
SOD	Superoxide dismutase
TBARS	Thiobarbituric acid reactive substances (lipid peroxidation)
